# Supplementary material for: Colonic Bacteria-Transformed Catechin Metabolite Response to Cytokine Production by Human Peripheral Blood Mononuclear Cells
Source: Biomolecules. 2019 Dec 5;9(12):830. doi: 10.3390/biom9120830 (PMC6995598; doi:10.3390/biom9120830)
Supplement: Supplementary file 1 [file biomolecules-09-00830-s001.pdf]

biomolecules

Supplementary Materials

Colonic Bacteria-Transformed Catechin Metabolite Response to Cytokine Production by Human Peripheral Blood Mononuclear Cells

Rajapandiyan Krishnamoorthy, Abdulraheem R. Adisa *, Vaiyapuri Subbarayan Periasamy, Jegan Athinarayanan, Subash-Babu Pandurangan and Ali A. Alshatwi *

Nanobiotechnology and Molecular Biology Research Lab, Department of Food Science and Nutrition, College of Food and Agriculture Sciences, King Saud University, Riyadh 11541, Saudi Arabia;

* **Correspondence:** rasheed.adisa@gmail.com (A.R.A.); alshatwi@ksu.edu.sa (A.A.A.); Tel.: +966543617783 (A.R.A.); Tel.: +966504236535, (A.A.A.)

In order to confirm the presence of separated Biotransformed metabolites fraction A (BTMFA) and B (BTMFB) HPLC-MS analysis were performed. The identification of Biotransformed metabolites were carried out on the basis of their MS spectral characteristics. The obtained chromatograms are shown in Figures S1 and S2. Accordingly, all detected peaks, present polyphenolic type spectra. The figure illustrates that the extraction of ion *m*/*z* corresponding to the metabolites of BTMFA and BTMFB were listed in the Table S1.

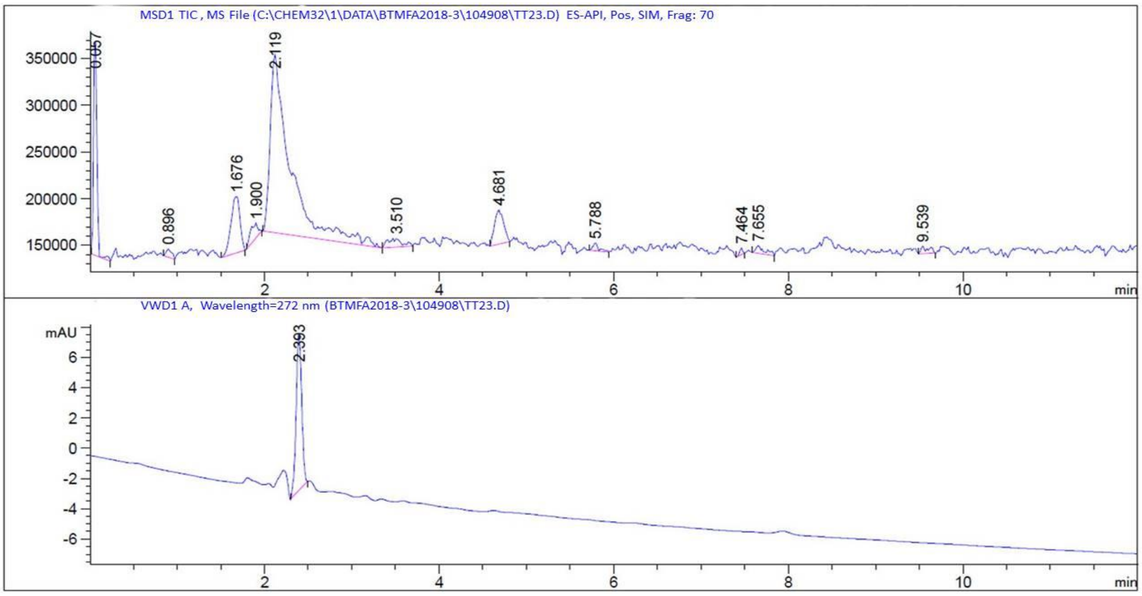

Figure S1. HPLC-MS chromatographic profile of BTMFA.

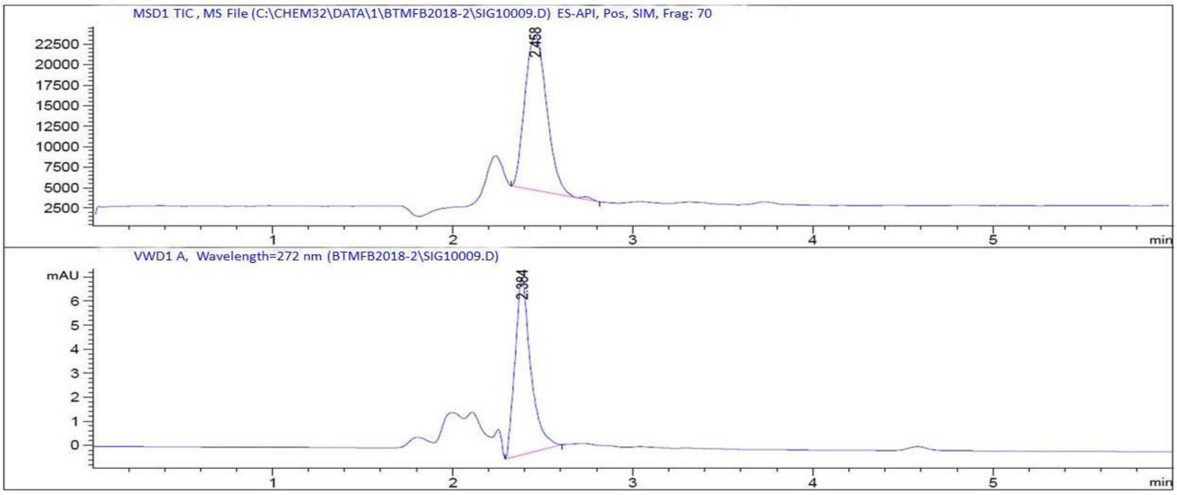

Figure S2. HPLC-MS chromatographic profile of BTMFB.

Table S1. The detected metabolites exact masses and observed **m/z** values in HPLC-MS.

S.no	BTMFA-Compounds	RT (min)	Exact Mass	Observed Peaks *m/z*
1	Dehydroquinic acid	3.510	190.152	190.8
2	4-ethylphenol	1.676	122.16	121.0
3	4-methoxyphenyl propan-2-ol	1.900	166.22	167.3
4	3-phenyl propionic acid	2.119	150.1745	149.2
5	2-phenoxyethanol	4.681	138.166	140.2
6	Benzene tricarboxylic acid,1,2-dimethyl ester	5.788	294.303	294.3
7	Catechol-1,4-benzenediol	0.896	110.112	110.9
8	Benzene-1,3,5-tris1-methylpropyl	7.464	246.438	243.1
9	3,5,7-trihydroxy-2H-chromen-2-one	7.655	194.142	195.8
10	4-hydroxyphenylpropionic acid	0.057	166.176	169.1
	**BTMFB-Compound**			
1	Dimethoxycinnamic acid	2.458	208.213	205.1

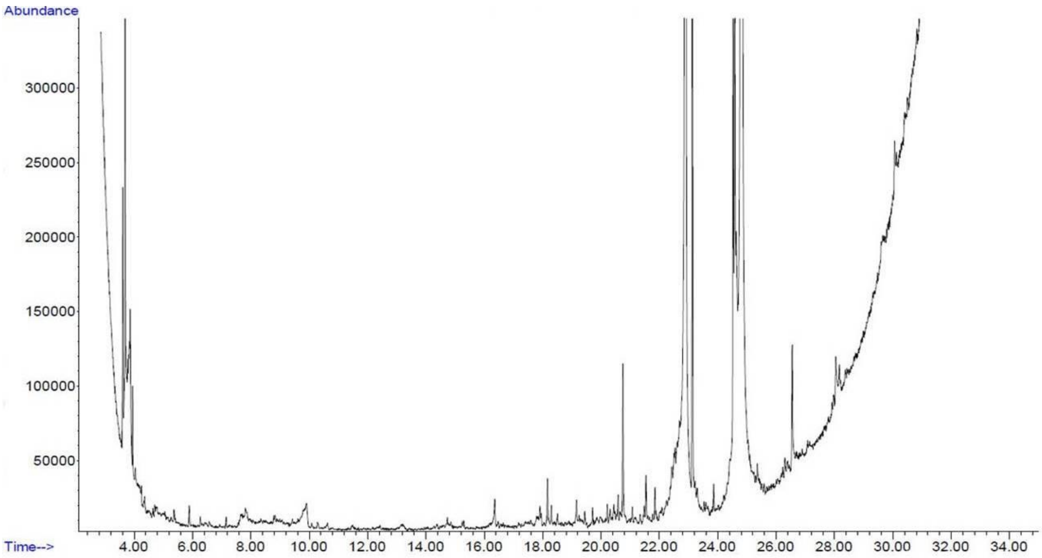

Figure S3. GCMS chromatographic profile of pooled fecal slurry.

According to GCMS analysis 40 metabolites were identified (Figure S3 and Table S2). All the metabolites present in the pooled fecal slurry which was used for the current experiment does not exist catechins or other form polyphenols. The identified metabolites normally found in human gut, which are produced during metabolic activity. Few metabolites are dietic origin particularly ethanoic, petanoic and hexanoic acids during fermentation of carbohydrates by bacteroides. Also, few aldehydes were found in the samples that are origin from hydrogenated form of carbohydrates in which carbonyl group has been reduced to be primary and secondary hydroxyl group.

Table 2. GCMS analysis metabolites detected in the pooled fecal slurry.

S.no.	Compound	RT	Mol. Weight	Mol. Formula
1	Palmitic Acid	3.45	256	$C_{16}H_{32}O_2$
2	1-Iodo-2-Methylundecane	3.61	296	$C_{12}H_{25}I$
3	1,3-Dioxolane-2-acetic acid, 2-methyl-	4.14	146	$C_6H_{10}O_4$
4	3-Hydroxybutan-2-one	5.36	88	$C_4H_8O_2$
5	2-Hexadecanol	5.36	242	$C_{16}H_{34}O$
6	3-Hydroxysuberic acid	5.89	190	$C_8H_{14}O_5$
7	Acetaldehyde	6.25	44	C_2H_4O
8	3-Methylglutarylcarnitine	7.15	289	$C_{13}H_{23}NO_6$
9	Butane-1,2,3,4-tetrol	7.67	122	$C_4H_{10}O_4$
10	Pentanedioic acid, 3-(hydroxymethyl)-	7.81	202	$C_9H_{14}O_5$
11	Butanal, 3-Methyl	8.75	86	$C_5H_{10}O$
12	Deoxycytidine	8.82	227	$C_9H_{13}N_3O_4$
13	Hexanal	9.06	100	$C_6H_{12}O$
14	Propanal, 2,3-Dihydroxy-, (*S*)-	9.43	90	$C_3H_6O_3$
15	Undecane	9.79	156	$C_{11}H_{24}$
16	Nonanal	10.08	142	$C_9H_{18}O$
17	Deoxyuridine	10.27	228	$C_9H_{12}N_2O_5$
18	Dl-Homoserine	10.63	119	$C_4H_9NO_3$
19	N-Methyl-2- Oxy Ethanamine	11.48	399	$C_{18}H_37NO_3SI_3$
20	Glycerophosphocholine	14.38	257	$C_8H_{20}NO_6P$
21	Hexanoic Acid, Butyl Ester	14.85	172	$C_{10}H_{20}O_2$
22	1-Pentadecene	16.34	210	$C_{15}H_{30}$
23	Butanal, 3-Methyl	17.14	86	$C_5H_{10}O$
24	Heptadecane, 2,6,10,14-Tetramethyl	17.39	296	$C_{21}H_{44}$
25	L-Cystathionine	17.91	222	$C_7H_{14}N_2O_4S$
26	1-Methylhistidine	18.14	169	$C_7H_{11}N_3O_2$
27	Glycerylphosphorylethanolamine	18.29	215	$C_5H_{14}NO_6P$
28	Docosane	18.50	310	$C_{22}H_{46}$
29	Pentanal	18.69	86	$C_5H_{10}O$
30	N-Pentadecanol	19.16	228	$C_{15}H_{32}O$
31	1-Hexadecanol	19.43	242	$C_{16}H_{34}O$
32	1-Octanol, 2-Butyl	20.19	186	$C_{12}H_{26}O$
33	Methyl Tetradecanoate	20.73	242	$C_{15}H_{30}O_2$
34	Nonanoic Acid, Methyl Ester	20.80	172	$C_{10}H_{20}O_2$
35	Hexadecanoic Acid, Methyl Ester	21.33	270	$C_{17}H_{34}O_2$
36	N-Acetylneuraminic acid	21.42	309	$C_{11}H_{19}NO_9$
37	Tridecanal	22.44	198	$C_{13}H_{26}O$
38	L-Palmitoylcarnitine	23.56	399	$C_{23}H_{45}NO_4$
39	3b-Hydroxy-5-cholenoic acid	23.61	374	$C_{24}H_{38}O_3$
40	Dl-Arabinitol	24.86	152	$C_5H_{12}O_5$
